# AGO2 involves the malignant phenotypes and FAK/PI3K/AKT signaling pathway in hypopharyngeal-derived FaDu cells

**DOI:** 10.18632/oncotarget.18047

**Published:** 2017-05-22

**Authors:** Yanhui Zhang, Baoxin Wang, Xinwei Chen, Weidong Li, Pin Dong

**Affiliations:** ^1^ Department of Otolaryngology Head and Neck Surgery, Shanghai General Hospital, Shanghai Jiao Tong University School of Medicine, Shanghai, China; ^2^ Department of Otolaryngology Head and Neck Surgery, Shanghai General Hospital, Shanghai, China; ^3^ Bio-X Institutes, Key Laboratory for The Genetics of Development and Neuropsychiatric Disorders (Ministry of Education), Shanghai Key Laboratory of Psychotic Disorders, and Brain Science and Technology Research Center, Shanghai Jiao Tong University, Shanghai, China

**Keywords:** AGO2, hypopharyngeal cancer, cell proliferation, FAK/PI3K/AKT signaling pathway

## Abstract

Argonaute 2 (AGO2) protein is usually overexpressed in various head and neck squamous cell carcinoma. However, the precise molecular mechanisms of AGO2 in hypopharyngeal cancer have not yet been clearly understood. Here we found the AGO2 expression in hypopharyngeal cancer tissues were generally higher comparing with that of the corresponding adjacent noncancerous epithelium tissues, and these were associated with the more aggressive clinicopathologic features and the poor clinical outcomes. Stable knockdown of AGO2 protein retarded cell proliferation, migration, invasion, arrested cell cycle and induced apoptosis. Meanwhile the knockdown also inhibited the FAK/PI3K/AKT signaling pathway in hypopharyngeal-derived FaDu cells. These findings suggested that *AGO2* gene might act as an oncogene which contributed to the tumorigenesis and progression, and has potential values for molecular diagnosis, clinical therapies and prognosis evaluation in hypopharyngeal cancer.

## INTRODUCTION

Hypopharyngeal cancer is a relatively rare but malignant heterogeneous disease in head and neck [1]. Most of them is hypopharyngeal squamous cell carcinoma (HPSCC). Although the quality of life for patients with hypopharyngeal cancer has been greatly improved by performing advanced and organ-preservation surgery and applying chemoradiotherapy over the past decades [2–4], the overall survival rate has barely increased due to local recurrence, regional lymph node and distant organs metastasis [5–7]. Alterations of oncogenes and tumor suppressors has been found on genetic and epigenetic levels in hypopharyngeal cancer, whereas conventional biomarkers are insufficient in early diagnosis and prognostic evaluations. Therefore, the genetic or molecular basis of hypopharyngeal tumorigenesis and progression are essential for efficient clinical practice.

Argonaute 2 (AGO2), the only member of the Argonaute family with an intrinsic endonuclease activity [8, 9], can be capable of directly cleaving mRNAs in a small RNA-guided manner [10], and also involves in the accumulation of mature miRNAs [11, 12]. Besides, AGO2 is the main component of RNA interference by way of constituting RNA-induced silencing complex (RISC), and functions as a RISC slicer to regulate RNAi efficiency and many other downstream cascades [8–10]. AGO2 can regulate neovascular formation by abnormally modulating angiogenic associated miRNAs [13] and most of the time can act as an oncogene in human carcinogenesis by promoting tumor growth [13–18]. AGO2 was expressed ubiquitously at a relatively low level in normal tissues, but up-regulated in different tumors, including those originated from breast, stomach, bladder and blood [13–15, 19–22]. However, the biological functions and molecular mechanisms of AGO2 in hypopharyngeal carcinoma have not been clearly understood. In this study, we evaluated the expression level and potential role of AGO2 in the progression of hypopharyngeal carcinoma.

## RESULTS

### Upregulation of AGO2 and correlation of its levels with clinical features in HPSCC

**Figure 1:**
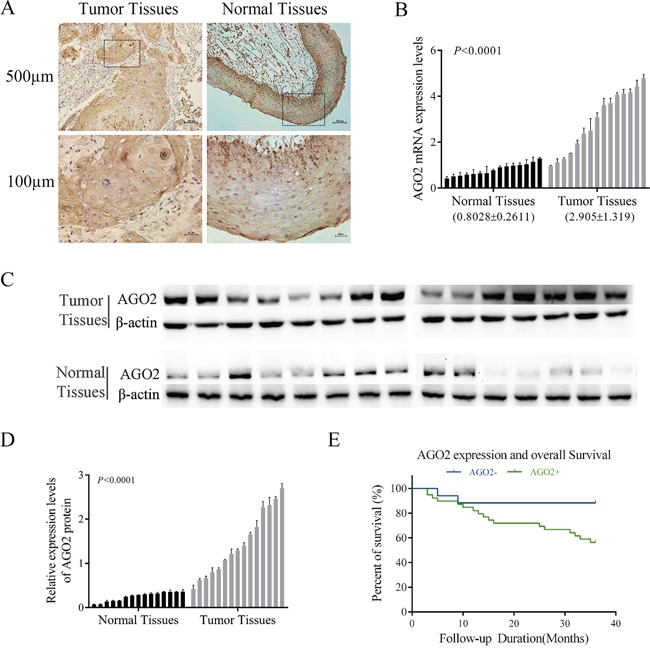
Expression of AGO2 in HPSCC **(A)** Representative images of AGO2 expression in HPSCC and adjacent noncancerous epithelium specimens (n=56) were obtained by IHC. AGO2 was positively detected in HPSCC (left, tumor tissues), diffusing cytoplasmic and part of nuclear staining, especially on the edge of the nests with pink keratin in the centers. AGO2 expressed in adjacent noncancerous epithelium was weakly detected (right, normal tissues). **(B, C, D)** AGO2 mRNA and protein expression data were acquired from 15 patients with HPSCC (tumor tissues) and 15 normal epithelium mucosae tissues of patients underwent uvulopalatopharyngoplasty (normal tissues). Expression levels of AGO2 mRNA were separately normalized to GAPDH. AGO2 protein expression levels were calculated by contrasting the gray levels of each band using ImageJ Software. The histogram represented the expression levels of AGO2 protein, which were normalized to β-actin. Error bars represented mean ± SD from three independent experiments. **(E)** Kaplan-Meier curve represented survival rate in patients with HPSCC, according to the expression levels of AGO2 (AGO2+, ≥3 score; AGO2-, <3 score). Blue, patients with negative expression of AGO2 (n=17, 3-year survival rate 88.24%); green, patients with positive expression of AGO2 (n=39, 3-year survival rate 56.41%; log-rank test, *P*=0.03).

To clarify the roles of AGO2 in the tumorigenesis and progression of hypopharyngeal carcinoma, the expression levels were assessed by immunohistochemistry among 56 pairs of formalin-fixed paraffin-embedded (FFPE) HPSCC tissues and corresponding adjacent noncancerous epitheliums. Among these specimens, 69.64% (39/56, Table 1) showed positive expression of AGO2, whereas the rest (30.36%, Table 1) showed negative. AGO2 was expressed highly in cancerous tissues, but not obvious in adjacent noncancerous epitheliums (Figure 1A). Real-time RT-PCR (Figure 1B) and western blotting analyses (Figure 1C and 1D) revalidated the higher expression of AGO2 in 15 pairs of fresh specimens newly acquired from hypopharyngeal cancer patients comparing with normal tissues from patients underwent uvulopalatopharyngoplasty for obstructive sleep apnea hypnea syndrome (two-tailed *t*-test, *P*<0.0001).

**Table 1:** Analyses of relationship between clinicopathologic features and AGO2 expression levels in HPSCC cases

Clinical Features	N (%)	AGO2 levels		*P* values
		Positive	Negative	
**Gender**				
Male	56 (100%)	39	17	0.330
Female	0			
**Age**				
< mean (57.54)	22 (39.29%)	17	5	0.380
≥ mean (57.54)	34 (60.71%)	22	12	
**Clinical stage**				
I-II	8 (14.29%)	2	6	0.007
III- IV	48 (85.71%)	37	11	
**Pathologic grade**				
High differentiation	18 (32.15%)	8	10	0.013
Moderate differentiation	32 (57.14%)	27	5	
Low differentiation	6 (10.71%)	4	2	
**Lymph node metastasis**				
N1˜3	47 (83.93%)	36	11	0.017
N0	9 (16.07%)	3	6	

The clinicopathologic features of the patients in our study were shown in Table 1: all the objects were males, and 39 out of 56 HPSCC tissues were positively stained with anti-AGO2 antibody. The average age was 57, ranged from 17 to 75. Most of the patients (85.71%) were in the terminal stage (III˜IV).

Further analyses were performed to elucidate the relationships between AGO2 expression levels and the clinicopathologic features or clinical outcomes of HPSCC. As shown in Table 1, the expression levels of AGO2 were significantly correlated with clinical stage (*P*=0.007), pathologic grade (*P*=0.013) and lymph node metastasis (*P*=0.017). And these correlations were also confirmed by Spearman correlation analysis (correlation coefficient: 0.837, 0.531, 0.684 respectively; Table 2). Kaplan-Meier survival analysis showed that the poor prognosis and worse overall survival in patients were associated with the higher AGO2 expression levels (*P*=0.03, Figure 1E).

**Table 2:** Spearman correlation analyses of AGO2 and clinicopathologic characteristics

Variables	AGO2 levels	
	Spearman correlation	*P* values
Clinical stage	0.837	0.007
Pathologic grade	0.531	0.013
Lymph node metastasis	0.684	0.017

There was a significant correlation between clinical stage and AGO2 positive staining (P<0.05, r=0.837). Besides, increased AGO2 expression was obviously associated with pathologic grade (P<0.05, r=0.531) and lymph node metastasis (P<0.05, r=0.684).

### Knockdown of AGO2 in hypopharyngeal-derived FaDu cells retarded cell proliferation, migration and invasion

**Figure 2:**
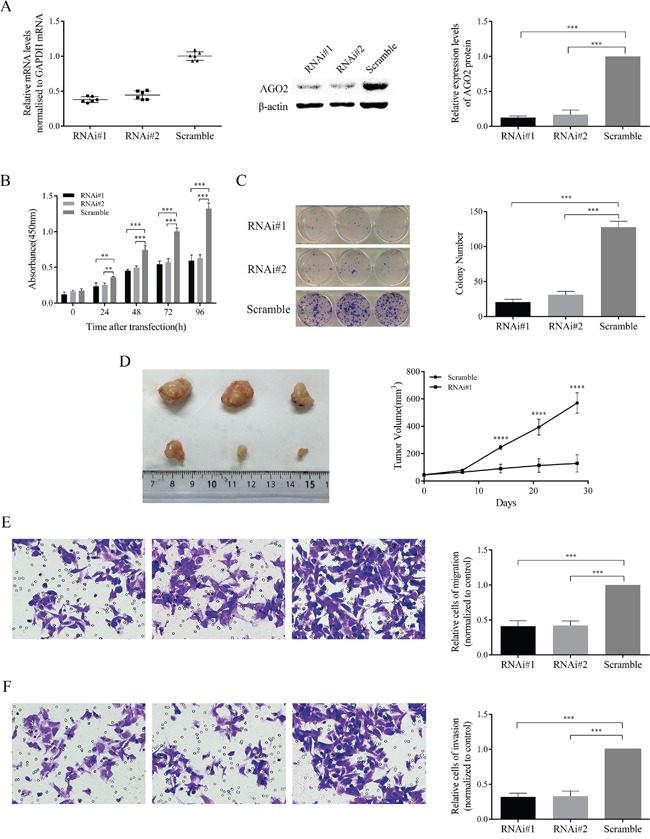
Inhibition effects of AGO2 knockdown on FaDu cells *in vitro* and *in vivo* **(A)** The expression levels of AGO2 mRNA and protein, significantly inhibited by lenti-shRNAs (RNAi#1 and RNAi#2) in comparison with control group, were determined by real-time RT-PCR and western blotting analyses. The expression levels of AGO2 mRNA were normalized to GAPDH. The protein quantities were determined by comparing the gray levels of each band using ImageJ Software, and normalized to control group. **(B)** In CCK-8 assay, the proliferation ability of stable transducted cells was obviously inhibited with time. **(C)** The left panel showed crystal staining of colonies. The histogram (right) represented the number of colonies formed. **(D)** The left panel showed tumors formed 4 weeks after inoculation. The right panel showed the growth curve. **(E, F)** The representative pictures showed cells migrated and invaded (left panels, 250μm). The histograms (right panels) represented the percentage of cells migrated and invaded, normalized to control group. *P<0.05, **P<0.01, ***P<0.001, ****P<0.0001.

To characterize the function of AGO2 in hypopharyngeal carcinoma, we created a cellular model by lentivirus-mediated stable knockdown of AGO2 expression in hypopharyngeal-derived FaDu cell lines. The AGO2 expression were significantly down-regulated verifying by real-time RT-PCR and western blotting analysis (Figure 2A). The lentiviral-infected FaDu cells showed a high percentage of GFP fluorescence, indicating a high efficiency and stability of transduction.

To investigate the possible knockdown effects on cell proliferation, we conducted CCK-8 cell viability assay and colony formation assay in stable transducted FaDu cells. Knockdown of AGO2 appeared to be significantly reduced the viability in various time periods (Figure 2B; *P*<0.001) and obviously decrease the colony number and size (Figure 2C; *P*<0.001) in FaDu cell lines. Tumor formation assay showed that knockdown of AGO2 caused remarkable inhibition on tumor growth in nude mice (Figure 2D; *P*<0.0001). Meanwhile, tumor cell migration and invasion abilities were also significantly inhibited *in vitro* in FaDu cells after down-regulation of AGO2 expression (Figure 2E and 2F; *P*<0.001).

### Knockdown of AGO2 in hypopharyngeal-derived FaDu cells arrested cell cycle and induced apoptosis

**Figure 3:**
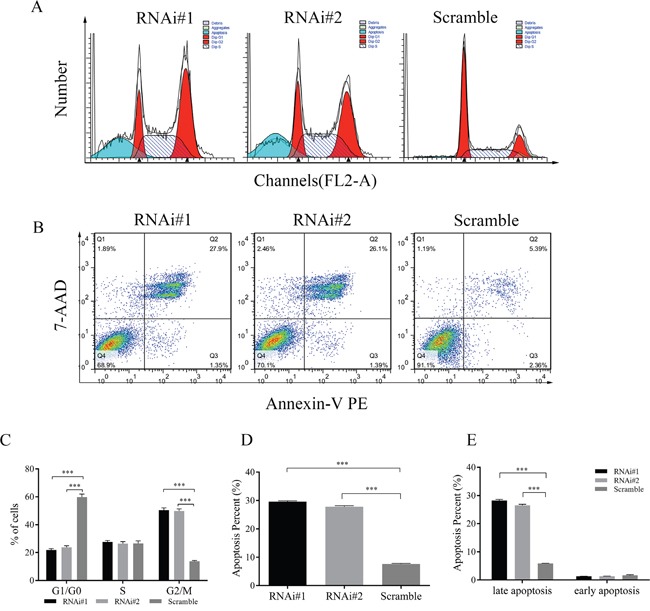
Effects of AGO2 knockdown on cell cycle and apoptosis of stable transducted FaDu cells were assessed by flow cytometry **(A)** Representative graphs showed the distribution of cell cycle. Compared with control group, AGO2 knockdown arrested cell cycle in G2/M phase. **(B)** Representative flow cytometry scatter diagrams of Annexin-V PE/7-AAD staining showed the distribution of viable, apoptotic and necrotic cells. Q1 (Annexin-V PE^-^/7-AAD^+^), Q2 (Annexin-V PE^+^/7-AAD^+^), Q3 (Annexin-V PE^+^/7-AAD^-^) and Q4 (Annexin-V PE^-^/7-AAD^-^) separately represented necrotic cells, late apoptotic and necrotic cells, early apoptotic cells, and viable cells. **(C)** The histogram represented the percentage of cells in G1/G0, S and G2/M phase. **(D)** The histogram suggested the quantitative results of the percentage of apoptosis in AGO2 knockdown cells and control cells. **(E)** The left side of the histogram showed the percentage of late apoptotic and necrotic cells, and the right side of the histogram is the percentage of early apoptotic cells (*P*=0.0673). *P<0.05, **P<0.01, ***P<0.001, ****P<0.0001.

To further explain the above phenotype of retarded cell proliferation in AGO2 knockdown FaDu cells, we predicted that AGO2 interruption could block the cell proliferation cycle and induced tumor cell apoptosis. Flow cytometric analysis with propidium iodide (PI) staining showed down-regulation of AGO2 arrested cell cycle in G2/M phase (Figure 3A). Statistical histogram revealed the population of cells decreased in G1/G0 phase, and increased in G2/M phase in AGO2 knockdown cells (Figure 3C; *P*<0.001). Fluorescence-activated cell sorting (FACS) analysis was performed on stable transducted FaDu cells double stained with Annexin V-PE/7-AAD (Figure 3B). The apoptosis rates in AGO2 knockdown cells were 29.58% and 27.83% respectively, whereas control cells showed only 7.57% apoptosis (Figure 3B and 3D; *P*<0.001). Moreover, there was statistically significant differences between AGO2 knockdown cells and control cells in late apoptosis (Figure 3E, left; *P*<0.001), while no significant differences could be found in early apoptosis (Figure 3E, right; *P*=0.067).

### Expression of Ki-67, cleaved Caspase-3 and E-cadherin changed in HPSCC and AGO2 knockdown xenograft

**Figure 4:**
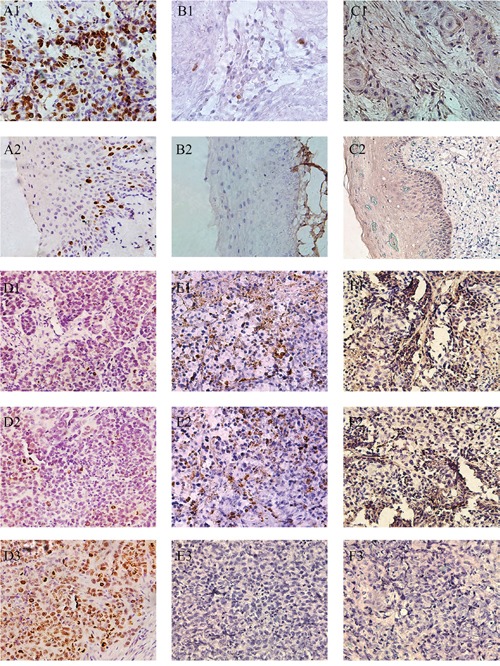
Representative pictures of IHC reflected the expression of Ki-67, cleaved Caspase-3 and E-cadherin in human hypopharyngeal tumor specimens and xenografts **(A1)** Ki-67 in HPSCC, **(A2)** Ki-67 in adjacent noncancerous epithelium, **(B1)** cleaved Caspase-3 in HPSCC, **(B2)** cleaved Caspase-3 in adjacent noncancerous epithelium, **(C1)** E-cadherin in HPSCC, **(C2)** E-cadherin in adjacent noncancerous epithelium, **(D1)** Ki-67 in RNAi#1 xenografts, **(D2)** Ki-67 in RNAi#2 xenografts, **(D3)** Ki-67 in Scramble xenografts, **(E1)** cleaved Caspase-3 in RNAi#1 xenografts, **(E2)** cleaved Caspase-3 in RNAi#2 xenografts, **(E3)** cleaved Caspase-3 in Scramble xenografts, **(F1)** E-cadherin in RNAi#1 xenografts, **(F2)** E-cadherin in RNAi#2 xenografts, **(F3)** E-cadherin in Scramble xenografts. Scale bars = 250μm.

To confirm these findings in FaDu cells, we analyzed the protein levels of Ki-67, cleaved Caspase-3 and E-cadherin by immunohistochemistry in 56 pairs of HPSCC and corresponding adjacent noncancerous epitheliums, as well as xenografts from 9 nude mice (Figure 4). The numbers of positive staining and staining intensities of Ki-67 in HPSCC were obviously more and stronger than the noncancerous epitheliums (73.21% vs 16.07%), especially in AGO2 overexpression tissues (84.62%, P<0.001). These results were reinforced in xenografts from nude mice with weak expression of Ki-67 in AGO2 knockdown tissues. It suggested that AGO2 could promote cell proliferation and tumor growth.

Meanwhile, sporadic cleaved Caspase-3 were observed in HPSCC with AGO2 overexpression (25.64%) as well as control xenografts, but a relatively higher expression of cleaved Caspase-3 were found in xenografts with AGO2 knockdown, which indicated AGO2 could suppress cell apoptosis and facilitate tumorigenesis.

Moreover, we found higher epithelial biomarker E-cadherin in AGO2 knockdown xenografts and noncancerous epitheliums, comparing with the lower levels in HPSCC tissues (26.79%), explained one of the possible pathophysiological mechanisms that AGO2 overexpression could promote epithelial-mesenchymal transition (EMT) and subsequent metastasis. These results further demonstrated that AGO2 exacerbated oncological behaviors in HPSCC, and knockdown of AGO2 inhibited these processes.

### FAK/PI3K/AKT signaling and other downstreams changed in HPSCC and AGO2 knockdown FaDu cells

**Figure 5:**
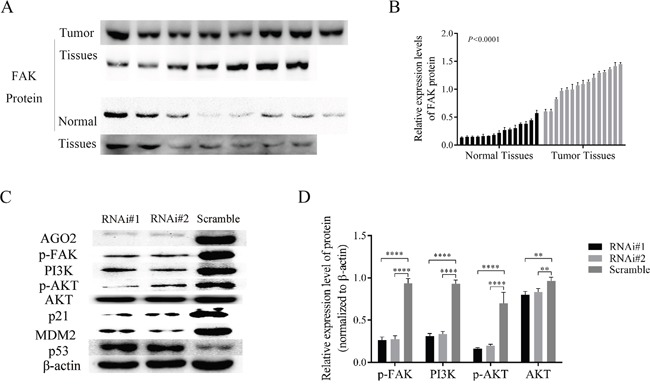
AGO2 knockdown inhibited FAK/PI3K/AKT pathway in stable transducted FaDu cells **(A)** The expression levels of p-FAK in HPSCC (tumor tissues) and adjacent noncancerous epithelium (normal tissues). **(B)** The histogram show the relative expression levels of p-FAK. **(C)** The expression of main proteins on FAK/PI3K/AKT pathway in stable transducted FaDu cells. **(D)** Knockdown of AGO2 inhibited the expression of p-FAK and the activity of PI3K/AKT signaling pathway. *P<0.05, **P<0.01, ***P<0.001, ****P<0.0001.

To dissect the molecular mechanisms of AGO2 in tumorigenesis, we performed western blotting analyses in previously described 15 cases of fresh hypopharyngeal carcinomas and 15 cases of normal epitheliums. The levels of FAK were up-regulated in tumor tissues (Figure 5A and 5B; *P*<0.0001), in accord with AGO2 expression pattern in the distinct tissues. Knockdown of AGO2 simultaneously attenuated the expression of phosphorylated FAK (p-FAK), also inactivated the PI3K/AKT signaling revealed by largely diminished the PI3K expression, p-AKT/ATK ratio, and downstream gene expression as MDM2/p53 or p21 (Figure 5C and 5D). These results implied that AGO2 promoted tumor growth and metastasis possibly through FAK/PI3K/AKT signaling pathway.

## DISCUSSION

As an oncogene located at chromosome 8q24 [23], AGO2 is usually expressed at a relatively low-level in normal tissues [15, 21, 22, 24], but frequently up-regulated in multiple human tumors [14, 15, 18, 20–22, 25]. Overexpression of AGO2 in colon cancer was positively associated with distant metastases [21]. Similarly, AGO2 was up-regulated in patients with urothelial carcinoma of the bladder, and accumulation of AGO2 implied higher tumor grading and poorer prognosis [22]. However, the relationship between AGO2 and hypopharyngeal carcinoma has not been well defined. In this study, we demonstrated that the expression of AGO2 was up-regulated in hypopharyngeal cancer at both mRNA and protein levels compared with adjacent noncancerous epithelium, which was in according with similar study in head and neck squamous cell carcinoma [26]. Up-regulation of AGO2 in hypopharyngeal cancerous tissues is significantly correlated with advanced clinical stage, higher tumor grade and poorer overall survival. Our study indicated that up-regulation of AGO2 might be recognized as a sign for more aggressive phenotypes and worse clinical outcomes.

Previous studies on AGO2 had proved its important roles in the developmental processes including neural tube closure, cardiac failure, and even embryonic lethal [9, 27–29]. In recent years, investigations were increasingly reported about the roles of AGO2 in tumorigenesis and progression. Overexpression of AGO2 was associated with high-risk myeloma and invasive hepatocellular carcinoma (HCC), while AGO2 knockdown caused a decline in viability and transferability of myeloma cell lines and HCC respectively [13, 15]. In addition, up-regulation of AGO2 promoted the activity of some miRNAs and the malignant phenotype of cervical carcinoma [30]. These data indicated that AGO2 acted as an oncogene in a variety of tumors. In this study, we found that knockdown of AGO2 in hypopharyngeal carcinoma cell lines resulted inhibitive effects on cell proliferation *in vitro* and *in vivo*. So we considered that this protein might also play an oncogenic role in hypopharyngeal cancer, and promised a potentially novel predictor.

Although knockdown of AGO2 has been proved to inhibit cell proliferation and tumor growth, the intrinsic molecular mechanisms are still unclear. It has been proved that the accumulated levels of AGO2 promoted tumorigenesis and metastases by binding to the promoter of FAK in HCC cells [15]. Interestingly, FAK is a core modulator of PI3K/AKT signaling mainly through forming complex with PI3K [31–33]. It is well known that AKT plays an essential role in downstream of the PI3K pathway, and is involved in multiple cellular processes [34–36] through dysregulation of signal transduction and subsequently aberrant activation or inactivation of important effectors, such as GSK3, p21 Cip1, p27 Kip1 and MDM2/p53 [37–41]. Besides, the PI3K/AKT signaling is usually hyperactive in multiple cancers, including hypopharyngeal cancer [42, 43], and promotes cell proliferation and tumor growth. Moreover, AGO2 has been found phosphorylated by AKT at S387 and indirectly inhibits tumor cell proliferation and tumor growth [44]. Thereby, we hypothesized and partially validated that knockdown of AGO2 retarded cell proliferation and tumor growth possibly through blocking the FAK/PI3K/AKT signaling in hypopharyngeal carcinoma.

p21 Cip1 and MDM2/p53 are key regulators of cell cycle and cell death downstream of AKT [41]. Inhibition of AKT pathway reduces the cellular levels of p21 and MDM2, which in turns increases the expression of p53, resulting in cell death and cell cycle arrest [45, 46]. Besides, MDM2 and p53 can be regulated at transcriptional levels mutually, and modulated by the AKT pathway at posttranscriptional level [47, 48]. In combination with our findings, the down-regulation of p21 and MDM2 and up-regulation of p53 probably resulted from the AGO2 knockdown. Taken together, our findings joined AGO2 with cell cycle and apoptosis regulation [49].

In conclusion, AGO2 is overexpressed in hypopharyngeal carcinoma, and up-regulation of AGO2 may be recognized as a valuable indicator for prognostic prediction. Knockdown of AGO2 can inhibit cell proliferation and tumor growth, decrease cell migration and invasion, arrest cell cycle and induce apoptosis. All these functions of AGO2 are firstly illuminated in hypopharyngeal cancer, and possibly mediated by FAK/PI3K/AKT pathway. While the details about the biological function and molecular mechanism of AGO2 in human hypopharyngeal cancer and the regulatory mechanism about AGO2 on the FAK/PI3K/AKT pathway need to be further investigated.

Besides, there are some limitations in our study. First, we didn't perform multivariate analysis due to the low number of clinical samples. Second, the regulatory mechanism of AGO2 on the FAK/PI3K/AKT pathway wasn't verified by up-regulating the expression of FAK. Third, most cases in our study are in advanced stage. So the results may not perfectly represent the actual early stage patients. Therefore, a larger-scale retrospective study including sufficient early stage cases is needed to confirm the biological functions and processes of AGO2.

## MATERIALS AND METHODS

### Patients and specimens

The clinical materials for research use were supported by the Ethics Committee of Shanghai General Hospital (Shanghai, China). 56 archived human HPSCC and adjacent noncancerous epithelium specimens were obtained from Pathology Department of Shanghai General Hospital. All cases were diagnosed clinically and histopathologically from Jan. 2010 to Dec. 2012. The patients are all males, aged from 17 to 75 (average age, 57). The clinical stage was determined according to the version 7 classic TNM-staging of the American Joint Committee on Cancer. The follow-up time for overall survival ranged from 3 to 36 months, during which 33.93% (19/56) of patients died. The fresh tumor and normal epithelium specimens, frozen in liquid nitrogen immediately after being resected, were separately acquired from 15 patients with HPSCC and 15 patients underwent uvulopalatopharyngoplasty since Jan. 2015 to Jun. 2016.

Those nominal variables, such as clinical stage, pathologic grade and lymph node metastasis, were transformed to ordinal categorical variables. The scoring criteria were as follows. The clinical stage was scored: 1 (Stage I), 2 (Stage II), 3 (Stage III) and 4 (Stage IV). The pathologic grade was scored: 1 (high differentiation), 2 (moderate differentiation) and 3 (low differentiation). And the lymph node was scored: 0 (no lymph node metastasis) and 1 (lymph node metastasis).

### Cell culture, vector construction and lentivirus infection

FaDu cell lines were procured from the Chinese Academy of Sciences (Shanghai, China) and cultured in Modified Eagle's medium (MEM; Gibco, life technologies, Grand Island, USA) supplemented with heat-inactivated (10%v/v) fetal bovine serum (FBS; Gibco, life technologies, Auckland, NZ) and (1%v/v) penicillin-streptomycin (P/S; Gibco, life technologies, Grand Island, USA). HEK-293T cells were kindly provided by Professor Weidong Li, and cultured in DMEM (Gibco, life technologies, Grand Island, USA) with FBS and P/S. All the cells were incubated at 37°C under 5% CO2.

To knock down AGO2, two short hairpin RNAs (shRNAs) sequences were separately designed and cloned into GV248 vectors. Then, the shRNA plasmids (shRNA#1 and shRNA#2) and control plasmid were transformed into Trans5α Chemically Competent Cell as suggested by the manufacturer (TransGen Biotech, Beijing, China). Positive transformants were selected, and plasmids from resistant colonies were extracted by EndoFree Maxi Plasmid Kit (TianGen Biotech, Beijing, China) and sequenced by HuaGene Biotech (Shanghai, China).

To acquire stable cell lines with AGO2 knockdown, HEK-293T cells (5×10^5^ cells/well) were seeded in 6-well plate. Three target plasmids together with two helper plasmids, psPAX2 and pMD2.G, were respectively transfected into HEK-293T cells using the classical calcium phosphate coprecipitation technique. 48h after transfection, about 85% of HEK-293T cells showed GFP. The mediums were collected and multiplicity of infection (MOI) was measured. FaDu cells were infected with lentivirus mediums and selected with 2μg/ml puromycin (Sigma-Aldrich, Saint Louis, MO, USA) for 2 weeks.

### real-time RT-PCR and western blotting analyses

Total RNA was extracted from human tissues and stable infected cell lines using the PureLink^®^ RNA Mini Kit (ambion, life technologies, USA). Reverse-transcription was conducted using PrimeScrip™ RT reagent Kit (Perfect Real Time, TaKaRa, Japan), and RT-PCR was performed using SYBR^®^Premix Ex Taq™ || (Tli RNaseH Plus, TaKaRa, Japan) on ABI PRISM 7500 Sequence Detection System (Applied Biosystems, Foster, CA, USA). All the process developed according to the manufacturer's protocol. Primers (Sangon, Shanghai, China) for RT-PCR were: AGO2, sense, 5’-ATCTTCTACCGCGACGGTGT-3’; antisense, 5’-GCTTGTCCCCCGCTCGT-3’; GAPDH, sense, 5’-GCACAGTCAAGGCCGAGAAT-3’; antisense, 5’-GCCTTCTCCATGGTGGTGAA-3’. The data were normalized to GAPDH and calculated by the 2-ΔΔCt method.

Proteins were extracted from human samples and stable infected cell lines, and measured using Bradford method to determine concentration. After electrophoresis in denature polyacrylamide gel, proteins were transferred to polyvinylidenedifluoride membranes (PVDF; Millipore, Billerica, MA, USA) and blocked with 5% milk. Then the membranes were immunoblotted with anti-AGO2 (Abcam- ab186733), anti-FAK (Y-397; Abcam-ab81298), anti-PI3K (Abcam-ab191606), anti-AKT (Abcam-ab179463), anti-p-AKT (S-473; Abcam-ab81283), anti-p21 (Abcam-ab109520), anti-MDM2 (Abcam-ab38618), and anti-p53 (Abcam-ab62376) antibodies. A rabbit monoclonal anti-β-actin antibody (CST-4970) was used as internal reference. Then membranes were incubated with species-specific secondary antibody (Invitrogen-31210) before developed with horseradish peroxidase (HRP; P90719, Millipore, Billerica, MA, USA).

### Cell proliferation and colony formation assay

Stable infected cells (3×10^3^ cells/well) were seeded on 96-well plates and cultured with 10μl CCK-8 (Sigma-Aldrich) 2h before detection. The optical density (OD) was measured at 0, 24, 48, 72, 96h after cells seeded by using microplate reader (BioTek, USA) at 450 nm. For colony formation assay, cells were seeded on 6-well plates (200 cells/well) and cultured for 2 weeks. The colonies were stained with crystal violet after being fixed with 4% paraformaldehyde (PFA). The two experiments were performed in triplicate respectively.

### Migration and invasion assays

Migration and invasion assays were performed by using Transwell chambers (Costar Corning, USA). Stable infected cells (5×10^5^ cells/well) were suspended in 200μl serum free MEM with 0.1% BSA and seeded into the upper chamber. 600μl MEM containing 10% FBS were added into bottom wells. After 24h, cells on the upper surface of the membrane were removed, and cells on the lower membrane surface were fixed with 4% PFA and stained with crystal violet. For the invasion assay, 80μg/ml of Matrigel (BD Biosciences, USA) were added to the upper chamber to form gel before cell seeding. Results were measured by counting cell numbers of five randomly selected non-overlapping fields by microscope (250μm). Three independent experiments were performed.

### Flow cytometry

Stable infected cells were trypsinized and processed according to the manufacturer's instruction (BD Biosciences, USA). For apoptosis assay, unstained cells, cells stained with Annexin-V PE alone, and cells stained with 7-AAD alone were separately used to set up compensation and quadrants. For cell cycle detection, cells were stained with PI (BD Pharmingen, USA). Flow cytometric analyses were performed on FACS Calibur instrument (BD Biosciences, USA). All the experiments were conducted in triplicate.

### Tumor formation assay

Stable infected cells (1×10^6^; RNAi#1 and scramble) were subcutaneously injected into each side of hind limb of male nude mice (4-6 weeks-old, n=3). All mice were housed and maintained under specific pathogen-free conditions. All animal experiments were agreed by the Animal Care and Use Committee of Shanghai General Hospital. We made every effort to reduce the mice for study and the discomfort they may suffer. Tumor size was measured weekly and tumor volume was calculated according to the formula 0.5×A×B2 (A: the base diameter of the tumor, B: the corresponding perpendicular value). All mice were sacrificed after 4 weeks. Tumors were excised, formalin-fixed and paraffin-embedded.

### Immunohistochemistry (IHC)

Paraffin-embedded human specimens and xenografts were deparaffined and rehydrated. All sections were heated in Tris-EDTA buffer (pH 9.0), treated with hydrogen peroxide, and incubated with primary antibodies against AGO2, Ki-67 (Abcam-ab92742), cleaved Caspase-3 (Abcam-ab32042) and E-cadherin (Abcam-ab76319) respectively. Then sections were incubated with species-specific secondary antibody (SV0004, Boster, Wuhan, China), developed with 3,3’-diaminobenzine (DAB, Invitrogen) and counterstained with hematoxylin.

IHC staining and scoring were determined by two clinical pathologists respectively according to the proportion of positively stained cells and staining intensity. The positively stained cells was scored: 1 (<10%), 2 (10-30%), 3 (30-60%), and 4 (>60%). The staining intensity was graded: 1 (light yellow), 2 (yellow to brown), 3 (brown), and 4 (deep brown). Staining index (SI) was determined by positive staining cells × staining intensity. The expression level of AGO2 was scored according to the SI. Score 1 and 2 were considered as negative, rest of the scores as positive.

### Statistical analysis

All statistical analyses were carried out using GraphPad Prism 5.0. Survival curves were plotted using the Kaplan-Meier method and analysed using the log-rank test. Spearman correlation analysis was used to study the relationship between AGO2 and clinicopathologic features of hypopharyngeal cancer patients. Unpaired *t* test was performed to analyze the data between two groups. ANOVA was applied to compare the differences between three groups. 0.05 was recognized as the standard *P* value for statistical significance. All statistical analyses were two tailed.
